# The Practice of Medicine

**Published:** 1907-03

**Authors:** 


### THE PRACTICE OF MEDICINE

---

Appears in many lights to one who travels and observes the customs of doctors of broad reputation. Some seem fully satisfied if they have properly diagnosed the disease, and leave some simple prescription rather as a placebo than as a real therapeutic agent. Such cases, they say, must die—whatever treatment is outlined; the other cases will live under any or no prescription if simply the fixed laws of hygiene are obeyed. Another class of able clinicians have apparently a sovereign remedy for all classes of diseases, and insist, to the minutest detail, upon the carrying out of every advice and the taking of medicines exactly as prescribed—to the very minute and drop. Another class treats their patients almost altogether symptomatically—without regard for the disease or accurate diagnosis. And so we observe in different centers different views as to value of drugs, or combinations; yet each claiming about an equal death rate or cure rate. Of course, we are not speaking of that class of so-called doctors who every day manifest ignorance in diagnosis, prognosis and the fundamental principles of therapeutics.

Why such records of success between different practitioners of eminence? Much observation of the facts leads us to believe that there is a much greater difference in results than the claims of some. A thorough knowledge of the disease in hand is unquestionably of paramount importance—its diagnosis and prognosis. But we sometimes apprehend that a strictly scientific diagnostician is not by any means a good therapeutist. He, too, readily satisfies himself with the natural history of the disease; and his almost inevitable fatal prognosis, under the do-nothing plan of treatment, results in error, when another practitioner who is familiar with therapeutic details takes charge of the case. Of course, we do not deny the vis medicatrix nature. But many lives, we are confident, are sacrificed because of the unfavorable prognosis. Cheerfulness and mental impression are undoubtedly wonderfully powerful instruments in the saving of life. Though the exact second of time for the administration of a properly prescribed medicine in a precise dose may not always be essential, such administration has its mental influence. It is a case of good engineering that brings success.

As for the purely "symptom" prescriber, there does not appear to be much in his favor when real disease confronts him. He may suit very well for a number of the unclassified common ailments every day seen in practice; but as a doctor who is able to add anything to the science of medicine he will be found wanting. Yet as these ailments are of such every-day occurrence where really the vis medicatrix nature does manifest itself, we need not refer further to this class of practitioners.

Let us leave the subject with our readers if we have succeeded in impressing the fact that the truly successful practitioner must be not only a good diagnostician but thoroughly acquainted with therapeutics as well.—Virginia Medical Semi-Monthly, February 8, 1907.

